# Financing STI testing among men in China: A mixed-methods study of pay-it-forward monetary donations

**DOI:** 10.1371/journal.pone.0342595

**Published:** 2026-02-13

**Authors:** Ye Liu, Ke Zhou, Lan Li, Gayed Salma, Thomas Fitzpatrick, Gifty Marley, Zixuan Zhu, Weiming Tang, Joseph D. Tucker

**Affiliations:** 1 University of North Carolina at Chapel Hill Project-China, Guangzhou, China; 2 Cambridge University NHS Foundation Trust, Cambridge, United Kingdom; 3 Department of Internal Medicine, University of Washington, Seattle, Washington, United States of America; 4 University of North Carolina at Chapel Hill, Chapel Hill, North Carolina, United States of America; 5 London School of Hygiene and Tropical Medicine, London, United Kingdom; Jiangsu provincial Center for Disease Control and Prevention, CHINA

## Abstract

**Aim:**

Many STI testing services are underfunded. Pay-it-forward is a strategy to support STI testing that asks participants to donate money to spur others to receive STI testing. To explore factors influencing monetary donations, we performed a secondary analysis of data from a randomized controlled trial that evaluated a pay-it-forward strategy to increase STI testing among men in China.

**Methods:**

We used a convergent parallel mixed-methods design to analyze data from the RCT and semi-structured interviews. Correlates of monetary donations were identified using logistic regression. Interview responses were analyzed using thematic analysis using charitable triad theory to explore donor, recipient, and organizational factors influencing donation behavior.

**Results:**

A total of 800 men received the pay-it-forward intervention. Overall, 139/718 (19%) made monetary donations, with a mean amount of 3.88 USD. The total value of all donations was 539.4 USD. At the donor level, donation behavior was associated with individual characteristics such as age, gender, and prior testing history. Donors who could identify with or visualize future recipients were also more likely to donate money. At the recipient level, income was not significantly associated with donation. At the organizational level, transparency and clear messaging enhanced trust and facilitated donations. An increased perceived risk of STI infection also motivated donations.

**Conclusion:**

Our findings highlight key factors driving donations in a pay-it-forward program. Transparent fund allocation and real-time donor feedback can enhance trust and participation.

**Clinical trial registration:**

ClinicalTrials.gov NCT05723263.

## Introduction

Sexually transmitted infections (STIs) remain a major public health challenge, especially [1]. in setting where access to testing services is often limited due to financing and structural constraints [2,3]. Fewer than 50% of countries provide STI testing for key populations, leading to missed diagnoses and ongoing transmission [1].

Given the high STI prevalence, improving testing uptake is a critical public health priority [4].However, traditional funding approaches for STI testing services—including fee-based systems and fully subsidized programs—have proven insufficient to meet population needs, particularly in resource-constrained settings [1]. While financial incentives have shown promise in increasing testing rates [4,5], their widespread implementation faces substantial barriers. Policymakers in low-resource settings frequently cite concerns about financial sustainability, potential for corruption in incentive distribution, health system weaknesses, and ethical considerations regarding frontline worker risks as key limitations [5]. These challenges occur against an unresolved backdrop of broader health financing debates regarding responsibility for public health service costs [6]. The tension between individual, governmental, and societal roles in funding essential health services remains a persistent dilemma in global health policy [7]. This context creates an urgent need for innovative financing strategies that are sustainable, feasible, and equitable.

The Pay-it-Forward (PIF) model operates by providing each participant with a subsidized or free STI test funded by a previous participant’s donation [8,9]. After receiving the test, participants are invited—without obligation—to donate any amount to support future individuals. This voluntary, cyclical mechanism integrates individual contribution, community solidarity, and institutional facilitation into a sustainable funding structure [10]. Previous studies have shown that pay-it-forward interventions effectively increase diagnostic test and vaccine uptake in China [11–16].

However, little is known about what motivates individuals to donate within PIF programs, as prior research has primarily examined testing uptake rather than the financial sustainability of the model. The specific drivers of giving in STI testing contexts remain underexplored. This limitation emphasizes the need to better understand donation determinants – both facilitators and barriers – to strengthen the approach's financial foundation. Addressing this knowledge gap is crucial for optimizing implementation strategies and ensuring the sustained public health impact of pay-it-forward.

To address this gap, we conducted a secondary analysis of data from the PIONEER trial—a multi-site, mixed-methods cluster randomized controlled trial (RCT) evaluating a pay-it-forward intervention to increase gonorrhea and chlamydia testing uptake among men in China [17]. The trial provided participants at public STI clinics and community-based organizations (CBO) the opportunity to donate money to support others to receive subsidized testing. The purpose of this study is to examine donor, recipient, and organizational factors associated with donating money to a pay-it-forward program based on mixed methods data.

## Methods

### Overview

Our secondary analysis used data from a convergent parallel mixed method study [17]. Our quantitative analysis identified correlates of contributing monetary donations as part of pay-it-forward. Semi-structured interviews explored participant donation decision-making processes.

### Setting

In the PIONEER study, four cities in Guangdong Province, China—Foshan, Zhuhai, Jiangmen, and Huizhou—received the pay-it-forward intervention. Each city had two trial sites: one sexual health clinic operated by a community-based organization and one public STD clinic operated by the local government. To be eligible for participation, individuals seeking care at the participating clinics had to be assigned male sex at birth, be at least 18 years old, and self-report having had sex without undergoing gonorrhea testing in the past 12 months. The pay-it-forward intervention provided chlamydia and gonorrhea testing as a gift, funded by donations from previous participants. The standard cost of the chlamydia and gonorrhea test was 21 USD (150 RMB). Participants were then invited to voluntarily contribute to support testing for subsequent participants.

### Quantitative data collection

From October 28th 2023 to May 24th 2024, 1200 men were enrolled. Men completed two sequential surveys to assess key outcomes. The enrollment survey captured baseline sociodemographic characteristics, sexual behaviors, STI testing history, and community engagement. Following intervention exposure, an exit survey measured four primary domains: monetary donation amounts; gratitude; community connectedness; and comprehension of the pay-it-forward mechanism. Comprehension items assessed whether participants recognized their test was funded by prior donations and whether they grasped that their contribution would support future participants. The gratitude scale used in this study was adapted from instruments used in prior PIF studies, with minor wording adjustments made for contextual suitability. The community connectedness scale was adapted from instruments used in prior PIF studies among sexual minority populations in China [10]. Both scales were pilot-tested among clinic clients to ensure cultural appropriateness and comprehension. Each gratitude item was scored on a 5-point Likert scale (1 = strongly disagree to 5 = strongly agree), with higher scores indicating greater gratitude. Community connectedness items were similarly scored on a 5-point Likert scale, and an aggregate score was constructed by summing all items. The two-item PIF comprehension measure assessed whether participants understood that their test was funded by previous donors and whether their own contribution could support future participants; each item was scored dichotomously (correct/incorrect). In addition, three survey items examined factors influencing donation willingness, with response options of agree, disagree, or neutral (S1 PIONEER Survey Instrument in S1 File).

### Quantitative data analysis

From June 20^th^ 2024, we analyzed donation patterns using a tiered analytical approach. First, descriptive statistics characterized overall donation behavior, with participants categorized dichotomously as donors or non-donors. A secondary analysis categorized participants into two groups based on donation amount: larger donations (more than $3.42 USD, 25 RMB) and smaller donations (less than $3.42 USD, 25 RMB). The threshold of $3.42 USD. This benchmark was selected because rapid HIV self-tests are one of the most commonly purchased community-based diagnostic tools among men in China, and no comparable market price exists for gonorrhea or chlamydia self-tests. Thus, the $3.42 threshold reflects a familiar and meaningful reference point for participants [18]. A sensitivity analysis using alternative cutoffs (median donation amount and quartile-based thresholds) yielded similar patterns of association, indicating that results were robust to the choice of cutoff. Chi-squared tests (χ² tests) and t-tests were used to compare characteristics and outcomes between participants who donated and those who did not. Subsequently, we constructed multivariable logistic regression approaches to identify independent predictors of donation behavior. For the primary outcome of donation participation (yes/no), approaches were adjusted for age, prior STI testing, intervention site, and community engagement scores. The secondary analysis of donation amount incorporated additional covariates, including marital status, awareness of funding sources, comprehension of fund allocation, and community connectedness scores.

### Qualitative data collection

Standardized semi-structured interviews were conducted with RCT participants. The interview guide including their reasons and feelings about donating or not, how they determined the donation amount, their perceptions of the recipients or organizers, and their views on funding sustainability(S2 PIONEER Semi-Structured Interview Guide in S2 File). Convenience sampling was used to recruit participants from study sites until thematic saturation was reached. In total, 27 men were included, purposively sampling men who have sex with men (MSM) and non-MSM individuals. All participants provided informed consent, participated anonymously, and received an incentive of $14 USD (100 RMB). The interviews, lasting between 35 and 65 minutes, were conducted either in person or via online calls. All interviews were audio-recorded and transcribed. Four trained interviewers with experience in qualitative research, as well as familiarity with the MSM population and STI care, conducted the interviews.

### Qualitative data analysis

**Fig 1:**
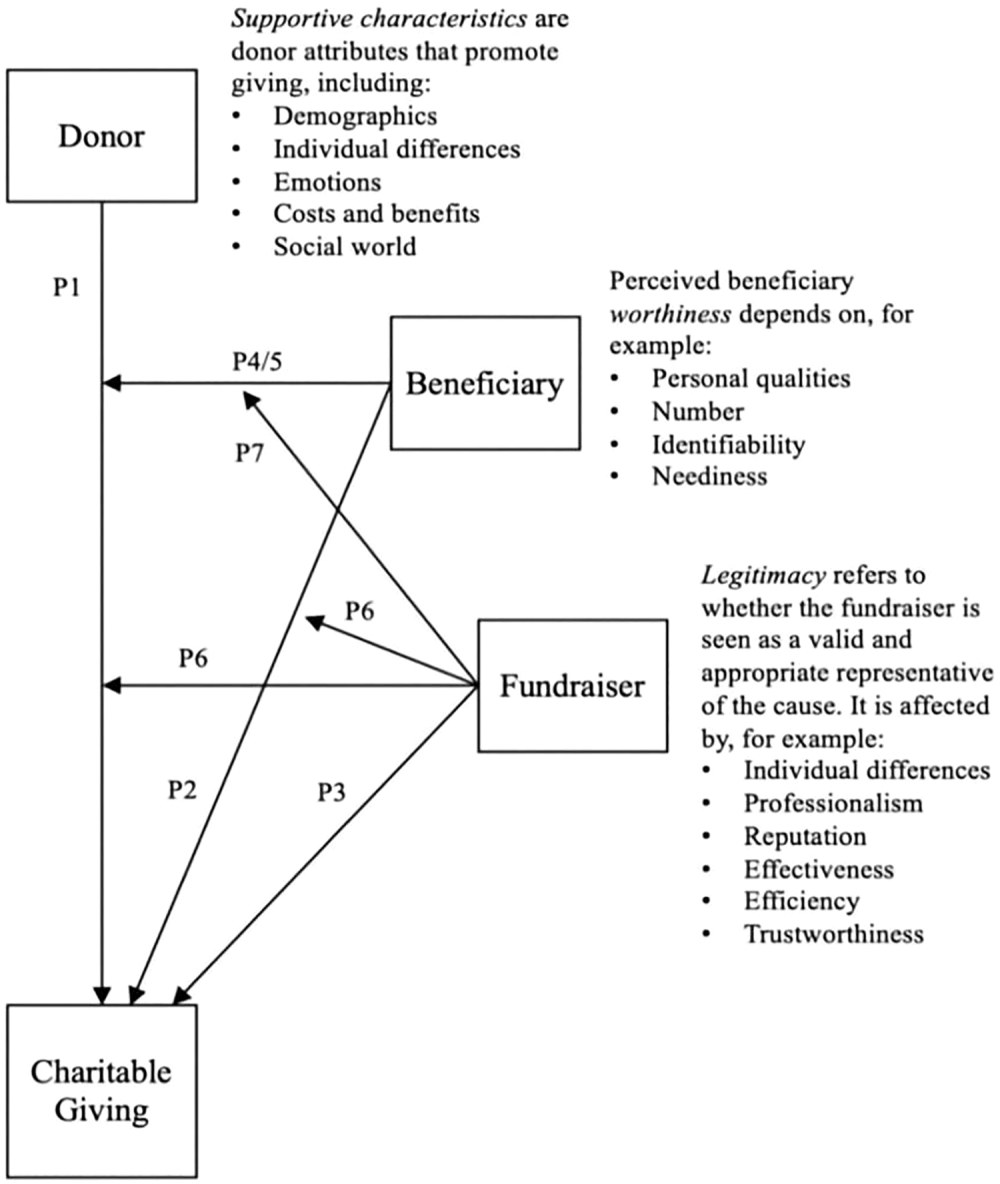
Charitable Triad Theory framework. This figure illustrates the three key actors in the pay-it-forward model—donors, recipients, and organizers—and their interactions influencing donation behavior.

From June 25^th^ 2024, textual data were analyzed using thematic analysis [19]. Coded using a hybrid deductive–inductive approach, guided by the Charitable Triad Theory [20]. All transcripts were coded in NVivo 12 software. Two trained qualitative researchers independently coded the transcripts. Both had prior experience in STI-related qualitative research and received additional training to ensure consistent application of the coding framework. Coding discrepancies were discussed in regular team meetings, and consensus was reached through iterative refinement of the codebook. The Charitable Triad theory examines triadic relationships among three key actors: the donor (i.e., individuals contributing money), the recipient (i.e., participants receiving subsidized STI testing), and the organizer (i.e., those implementing the intervention, including the research team). This framework explores the characteristics and interactions of these three actors and how they influence donation behavior (Fig 1). All men received free STI testing, with the intervention intentionally creating a dual role—every donor was also a past recipient. This design aimed for an exploration of motivation from two perspectives: (1) reflecting on their initial experience as recipients and (2) considering how their donation could support future beneficiaries.

### Ethics statement

This study was approved by the ethics review committee at the Southern Medical University Dermatology Hospital (approval no.IIT-2023–134) and the institutional review board at the University of North Carolina at Chapel Hill (approval no.21–1667). Informed written and oral consent obtained from all participants prior to enrollment in the study. All participants also provided explicit consent for their submitted data to be used in subsequent analyses. Additional information regarding the ethical, cultural, and scientific considerations specific to inclusivity in global research is included in the Supporting Information (S3 Inclusivity Checklist in S3 File)

## Results

**Fig 2:**
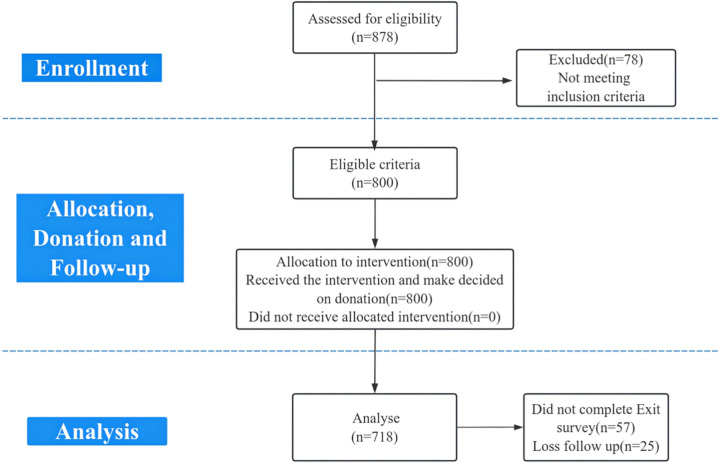
Adapted consort flow diagram showing study recruitment process. This figure shows the adapted participant flow and consent process for the PIONEER trial.

Among the 1,200 eligible men enrolled in the PIONEER trial, 800 men joined from sites assigned to the pay-it-forward intervention(S4 Dtata). 718/800 men (89.8%) completed both enrollment and exit surveys (Fig 2).

As shown in Table 1, participants had a mean age of 35 years; 57.5% (413/718) were married, 48.9% (351/718) held a bachelor’s degree or higher, and 41.9% (301/718) earned more than $8,280 USD per year. More than half of participants identified as MSM (51.0%, 366/718), and 44.4% (319/718) reported HIV, syphilis, or hepatitis C virus (HCV) testing in the past year. 94/718 (13.1%) of men received a gonorrhea test in the past and 101/718 (14.1%) of men received chlamydia test.

**Table 1:** Socio-demographic characteristics, sexual behaviors and previous testing of the participants enrolled in the pay-it-forward arm (N = 718).

	Variable	Overall	Non-donors	Donors	χ²/t	P value
		N = 718 N (%)	n = 579 N (%)	n = 139 N (%)		
Age*	Mean±SD	36 ± 13	36 ± 13	35 ± 10	0.17	0.768
	≤30 years old	420 (58.5)	349 (60.3)	71 (51.1)	3.54	0.06
	>30 years old	298 (41.5)	230 (39.7)	68 (48.9)		
**Clinic type**	CBO-led clinic	340 (47.4)	287 (49.6)	53 (38.1)	5.43	0.02
	Public clinic	378 (52.6)	292 (50.4)	86 (61.9)		
**Marital status**	Ever married	305 (42.5)	239 (41.3)	66 (47.5)	1.52	0.217
	Never Married	413 (57.5)	340 (58.7)	73 (52.5)		
**Level of Education**	Below high school	191 (26.6)	148 (25.6)	43 (30.9)	2.13	0.345
	High school	176 (24.5)	141 (24.4)	35 (25.2)		
	Bachelor's degree	351 (48.9)	290 (50.1)	61 (43.9)		
**Annual income/USD**	≤4956	202 (28.1)	170 (29.4)	32 (23.0)	2.36	0.308
	4968 ~ 8280	215 (29.9)	172 (29.7)	43 (30.9)		
	>8280	301 (41.9)	237 (40.9)	64 (46.0)		
**Sexual Identity**	MSM	366 (51.0)	305 (52.7)	61 (43.9)	3.12	0.077
	Non-MSM	352 (49.0)	274 (47.3)	78 (56.1)		
**Previous STDs ever tested**	HIV	386 (53.8)	318 (54.9)	68 (48.9)	3.13	0.6798
	Syphilis	366 (51.0)	300 (51.8)	66 (47.5)		
	HCV	323 (45.0)	263 (45.4)	60 (43.2)		
	HBV	319 (44.4)	255 (44.0)	64 (46.0)		
	Gonorrhea	94 (13.1)	73 (12.6)	21 (15.1)		
	Chlamydia	101 (14.1)	77 (13.3)	24 (17.3)		
**Factors associated with donation willingness**						
**Perceived donation obligation**	Disagree	40(5.11)	40(6.1)	–	–	
	Neutrality	96(12.26)	84 (13)	12(7.7)		
	Agree	647(82.63)	505(78.1)	142(92.2)		
**Self-prioritization reluctance**	Disagree	158(20.18)	120(18.5)	38(24.6)		
	Neutrality	186(23.75)	159(24.6)	27(17.5)		
	Agree	439(56.07)	350(54.1)	89(57.7)		
**Financial constraint reluctance**	Disagree	110(14.05)	74(11.4)	36(23.3)		
	Neutrality	179(22.86)	147(22.7)	32(20.7)		
	Agree	494(63.09)	408(63.1)	86(55.8)		

Note: SD = Standard Deviation; CBO = Community-based Organization; STD = Sexually Transmitted Disease; Ever been married = included currently married, divorced, widowed, and separated; USD = United States Dollars; MSM = Men have sex with other men; HIV = Human Immunodeficiency Virus; HCV = Hepatitis C Virus; HBV = Hepatitis B Virus.

*Age is presented both as a continuous variable (mean ± SD) and as a categorical variable (≤30 vs. > 30 years) to facilitate comparison with prior PIF studies.

Following the pay-it-forward intervention, 94.2% (676/718) completed gonorrhea and chlamydia testing. In terms of the factors associated with their donation willingness, 82.6% of participants felt a sense of obligation to give back once they learned that the donation came from others, while 56.1% of participants agree that their reluctance to donate stemmed from wanting to spend money on themselves rather than others and 63.1% of participants agree that they reluctant to donate due to financial constraints. Table 2 summarizes the number and amount of monetary donations. Among all 718 men, 139 (19%) men made a monetary donation, with a mean donation amount of 3.88 USD. The cumulative value of all donations was 539.4 USD. Among men who donated, 57 (41%) contributed 1.40 USD and 29 (20.86%) contributed 7 USD. The largest individual donation was 21 USD.

**Table 2:** Descriptive summary of donation amounts received in the pay-it-forward intervention, stratified by STI testing acceptance.

Variable	Total, N	Donate, N	Total	Average
			Donation amount	Donation amount
**All participants**	718	139	3,924.48 RMB (539.471USD)	28.23 RMB (3.88 USD)
**Received STI testing**	676	134	3,794.48 RMB (521.61USD)	28.32 RMB (3.89 USD)
**Not received STI Testing**	42	5	130 RMB (18.40 USD)	26 RMB (3.68 USD)

The qualitative component consisted of in-depth interviews with 27 participants (24 conducted in person and 3 via online platforms). Donations were more common among participants recruited for semi-structured interviews. Collectively, the 27 interviewees donated 79.73 USD (580 RMB), with a mean donation amount of 3.80 USD, and donation amounts ranged from 1 to 21 USD. These findings were systematically integrated with quantitative results. We identified donor, recipient, and organizer factors that facilitated monetary donations as part of the pay-it-forward intervention.

### Donors

The gratitude score was strongly associated with participants’ donation behavior. As the gratitude scale score increased, individuals were more likely to donate (OR: 1.59, 95% CI: 1.24–2.08). Variables included in the multivariable logistic regression model were selected a priori based on theoretical relevance and prior literature on PIF studies, rather than solely on univariate significance. Age, income, prior STI testing, and community engagement were retained as potential confounders. After adjusting for potential confounders, this association remained significant (aOR: 1.61, 95% CI: 1.24–2.13) (Table 3). Most donors viewed their contributions as a means of giving back, helping to prevent STI among subsequent men and help others.

**Table 3:** Factors associated with donation rates.

Variables	OR	OR 95% CI		aOR	aOR 95% CI	
**Clinic type**						
Public STD Clinic	Ref			Ref		
CBO-led Clinic	0.63*	0.43	0.92	0.60*	0.36	0.97
**Perceived source of funding**						
Perceived funding from previous participants	Ref			Ref		
Perceived funding from other sources (government, CDC, hospital, CBO)	1.11	0.76	1.63	1.93*	1.21	3.09
**Perceived use of funds**						
Supporting future participants	Ref			Ref		
Supporting institutional efforts (e.g., government, CDC programs)	0.07*	0.001	0.50	0.40*	0.25	0.64
Unaware of donations	0.57*	0.38	0.83	0.05*	0	0.36
**Gratitude score**	1.59*	1.24	2.08	1.61*	1.24	2.13

+Adjusted for age groups, income group, previous STI testing, STI testing acceptance, and community engagement score.

*P values<0.05.

In semi-structured interviews, donors identified several personal characteristics that influenced their donation behavior. Income level emerged as a key determinant of donation amounts. The flexibility of the pay-it-forward approach allowed donors to contribute within a self-determined range, mitigating the psychological burden associated with perceived social expectations. As one donor explained, “I don’t want to take advantage of free testing, but I also don’t want to overextend myself by donating beyond my means” (MSM). Most donors perceived small contributions as acceptable, often comparing them to the cost of “buying a cup of milk tea,” a popular and affordable beverage in China priced between 15 and 30 RMB (approximately 2–4 USD). This mental accounting framed the donation as a reasonable exchange for the cost of the test, reinforcing a sense of fairness in their contribution.

The perceived risk of STI infection influenced donation amounts in the pay-it-forward strategy. Among participants engaging in high-risk sexual behaviors (e.g., multiple partners, condomless sex, or casual encounters; n = 6), four men reported heightened health anxiety as a motivator for getting tested, and three contributed donations exceeding $14 USD. This pattern suggests a form of compensatory reciprocity, wherein the anticipation of health benefits reinforces a sense of obligation to give back. As one participant stated, “Since I might have benefited from the service, donating felt like a way to balance the scales” (Donor, MSM).

### Recipients

Regression analysis revealed that participants who perceived their testing was funded by external sources (e.g., government, CDC, hospital, or CBO) had nearly twice the odds of donating compared to those who attributed funding to a previous participant (aOR: 1.93, 95% CI: 1.21–3.09). Conversely, those unaware that the pay-it-forward model supported future recipients had lower odds of donating (aOR: 0.05, 95% CI: 0–0.36). Interestingly, men who believed donations would be used for institutional efforts (e.g., government or CDC programs) had 50% lower odds of donating than those who thought funds would support future participants (aOR: 0.40, 95% CI: 0.25–0.64) (Table 3).

In semi-structured interviews, participants emphasized how the pay-it-forward intervention fostered donation through shared identity alignment and perceived social impact. Within the MSM community, donors expressed greater willingness to contribute when they identified with recipients and perceived the strategy as sustainable. As one donor stated, ‘I’m happy to support it once I learned that the initiative would have ongoing follow-up efforts’ (MSM), highlighting a desire for extended program impact. Some MSM participants further believed that this approach could reduce STI stigma, encouraging larger donations (Donor, MSM). Among non-MSM participants, the pay-it-forward approach was valued for addressing sensitive issues related to STI testing by alleviating the discomfort associated with seeking assistance. They emphasized its role in facilitating early detection, which could help curb disease transmission. Additionally, they noted that cumulative donations not only funded testing for others but also fostered broader community support (non-donor, non-MSM).

Social desirability and moral pressure played a significant role in shaping donation decisions by fostering an emotional connection with recipients. Donors often perceive their contributions as a means of reciprocating kindness. As one donor explained, “Since I received free testing and even a subsidy after the test, I would feel guilty if I didn’t donate—especially knowing that my testing fee came from donations from previous participants” (Donor, MSM). This also contributed to 82.6% of participants acknowledging the statement: “Once I learned that the donation came from others (knowing that the local community cared about me and contributed to my test), I felt an obligation to give back to others.”

### Organizer

The clinical setting in which STI testing was conducted was associated with both the proportion of men who donated and the donation amount. After adjusting for relevant parameters, men tested at public STD clinics had lower odds of donating compared to those tested at CBO-led clinics (aOR: 0.6, 95% CI: 0.4–1.0) (Table 3). Furthermore, participants at CBO-led clinics were significantly more likely to make larger donations than those at government-run STD clinics (aOR: 7.89, 95% CI: 2.82–24.68)(Table 4), even after adjusting for relevant factors. The wide confidence intervals observed in the donation-amount model reflect the relatively small number of participants who made larger donations (n = 45). All covariates listed in the footnote were included in the adjusted model; however, model stability was checked by refitting the model with penalized likelihood estimation, and the direction and magnitude of effect estimates remained consistent.

**Table 4:** Factors associated with donation amount.

	≤25 RMB**	>25 RMB	OR	aOR*
	(N = 94)	(N = 45)		
***Clinic type***				
Public STD Clinic	71 (75.5%)	15 (33.3%)	Ref	Ref
CBO-led Clinic	23 (24.5%)	30 (66.7%)	6.17	7.894
			(2.84–13.44, p < .001)	(2.824–24.68, p < .001)
***Annual income/USD***				
≤4956	26 (27.7%)	6 (13.3%)	Ref	Ref
4968 ~ 8280	35 (37.2%)	8 (17.8%)	0.687	4.024
			(0.19–2.52, p = .566)	(1.33–13.57, p = .013)
>8280	33 (35.1%)	31 (68.9%)	4.11	3.89
			(1.65–10.22, p = .002)	(1.21–12.55, p = .023)

*Adjust for married status, acceptance of STI testing, perceived source of funding, perceived use of funds, STI testing history, community engagement score, gratitude score and community connectedness score.

**25RMB ≈ 3.42 USD.

In semi-structured interviews, participants highlighted the influence of organizers on their donation decisions. In contrast, CBO-led clinics were described as facilities that ‘often provide free testing services’ (Non-donor, MSM), which may reduce participants’ expectations of payment. However, it is interesting that the strong connection between CBO-led clinics and the MSM community may enhance willingness to make larger donations.

The decision to donate was closely aligned with donors’ beliefs in the principles of kindness. When fundraising efforts were perceived as credible, transparent, and sustainable, individuals were more inclined to contribute. One donor articulated this by stating, ‘Notify me when my donation gets used’ (MSM). In contrast, many non-donors expressed hesitation due to a lack of clarity regarding the recipients and the usage of the funds. As one non-donor remarked, ‘I might be more inclined to donate to a specific person or tangible cause’ (Non-donor, MSM). Additionally, for marginalized groups like MSM, structural stigma heightened doubts about the sustainability of such initiatives. One participant expressed: ‘Since our community isn’t openly discussed here, people always doubt how long an MSM-focused testing strategy can last’ (Non-donor, MSM).

## Discussion

This study identified complex interactions among donors, recipients, and organizers that influenced monetary donations as part of a pay-it-forward approach. Quantitative analysis identified three key drivers of donation: donor, recipient identifiability, and the type of clinic. Qualitative findings further highlighted that individuals’ perceived STI risk and perceived transparency of fund usage were central to their donation decisions. Our study extends the existing literature by applying a mixed-methods approach to understand donations in an innovative financing program in a middle-income country setting. This study informs new strategies for financing public health services in resource-limited settings.

Many countries rely on short-term donor-supported STI screening programs with uncertain long-term financial sustainability [1]. Evidence from HIV self-testing programs also shows that secondary distribution models can expand reach but do not inherently provide mechanisms for financial sustainability [21]. Our findings provide insight into how pay-it-forward compares with other financing strategies for STI testing, such as user-paid testing, free testing, and secondary distribution of self-testing kits. Compared to user-paid services, pay-it-forward introduces a social dimension of giving and receiving that may reduce perceived transactional barriers. In contrast to free testing programs, which have been shown to effectively increase uptake, particularly among key populations [22], pay-it-forward adds a layer of sustainability by encouraging user contributions. Secondary distribution approaches, often used for HIV self-testing, rely on peers to expand reach but do not involve financial reciprocity [3]. Taken together, our findings suggest that pay-it-forward can complement these strategies by tapping into community solidarity and shared responsibility for health services.

The overall rate of monetary donations observed in our trial was much lower than previous pay-it-forward studies [11]. This discrepancy may reflect a shifting landscape in STI testing services, namely, an increasing availability of free or subsidized services. Free STI testing through CBOs has become increasingly widespread. In several cities, targeted free screening programs for gonorrhea and chlamydia have become more common as part of local public health surveillance efforts. Therefore, the perceived need for individual financial contribution may diminish. As pay-it-forward gradually transitions from a novel research-based intervention into a broader public health strategy, the perceived necessity of donation may decline. This evolution suggests that the rationale for donating in pay-it-forward may become less salient if free programs are widely available.

Our mixed-methods analysis revealed that donation behavior was not associated with income level. While counterintuitive, this finding echoes debates in behavioral economics, where evidence on the income–donation link remains mixed. Large-scale studies across countries have shown no consistent relationship between wealth and prosocial giving [23], while others argue that high inequality may reduce generosity among the wealthy [24]. Conversely, some research suggests that in highly unequal societies, affluent individuals may display greater pro-sociality due to heightened social awareness [25]. Our study adds a unique case from a middle-income context, pointing to the importance of cultural and structural moderators in shaping giving behavior.

Transparency emerged as a critical factor influencing donor trust. Both donors and non-donors emphasized the need for clear communication about fund usage. Participants expressed a desire to make meaningful contributions and viewed transparency as a means to ensure their donations had real impact. These findings align with existing evidence that transparency enhances trust and participation [26], and that feedback mechanisms, such as displaying how donations are used, can activate feelings of reciprocal altruism [11,27]. Ensuring transparency in fund allocation is therefore crucial to the sustainability of pay-it-forward strategies. Participants who were unable to explain how donated funds were used were more likely to donate. Rather than reflecting prosocial calculation, this pattern may indicate that some donors relied on low-effort, heuristic decision-making—donating impulsively or out of momentary emotion without fully processing how the funds would be used. Such low-effort decisions are consistent with “heuristic giving,” [28] where individuals respond to simple cues or momentary affect rather than engaging in detailed cognitive evaluation. Further research is needed to explore this phenomenon and disentangle the emotional versus rational drivers of donation within social innovation programs.

Several limitations warrant consideration. First, this was a secondary analysis and was limited by the available dataset, which lacked key variables such as medical history or chronic disease status. Second, participants in the qualitative study were more likely to be those who had received STI testing and were recruited from CBOs. This approach may introduce selection bias and not capture barriers to testing. Third, people who participated in the qualitative study likely understood pay-it-forward more than the average participant, making it difficult to understand routine services. Forth, all study sites were located within Guangdong Province, the findings may not fully generalize to settings with different STI service infrastructure or cultural norms.

This study offers new perspectives on how pay-it-forward mechanisms operate in real-world STI testing programs. It demonstrates that contextual factors—such as clinic type, perceived fund transparency, and donor-recipient dynamics—can significantly shape giving behavior. As health systems seek to develop sustainable, community-engaged financing approaches, pay-it-forward strategies offer a promising option. Future efforts should prioritize clarity in messaging, real-time feedback on donation use, and context-specific adaptation to maximize impact.

## Conclusion

Our findings have important implications for research and program design. Future studies should investigate how different healthcare settings influence donation behaviors. Contrary to assumptions that low-income individuals need more encouragement to donate, our data suggest that they already actively participate in pay-it-forward approaches. Therefore, future research should focus on strategies to elicit larger donations from higher-income participants, such as tailored messaging or recognition mechanisms. In terms of program implementation, ensuring transparency through clear communication about fund allocation and impact remains essential. Providing real-time updates on how contributions support others may further enhance trust and engagement. Strengthening these elements will be critical for optimizing the sustainability and scalability of pay-it-forward interventions.

## Supporting information

S1 File PIONEER Survey Instrument. (DOCX)

S2 File PIONEER Semi-Structured Interview Guide. (DOCX)

S3 File Inclusivity Checklist. (DOCX)

S4 File Data. (XLSX)

## References

[1] WHO Global progress report on HIV, viral hepatitis and sexually transmitted infections 2021.

[2] Unemo M, Bradshaw CS, Hocking JS, de Vries HJC, Francis SC, Mabey D, et al. Sexually transmitted infections: challenges ahead. Lancet Infect Dis. 2017;17(8):e235–79. doi: 10.1016/S1473-3099(17)30310-9 28701272

[3] Tsadik M, Berhane Y, Worku A, Terefe W. The magnitude of, and factors associated with, loss to follow-up among patients treated for sexually transmitted infections: a multilevel analysis. BMJ Open. 2017;7(7):e016864. doi: 10.1136/bmjopen-2017-016864 28716795 PMC5726144

[4] Lee R, Cui RR, Muessig KE, Thirumurthy H, Tucker JD. Incentivizing HIV/STI testing: a systematic review of the literature. AIDS Behav. 2014;18(5):905–12. doi: 10.1007/s10461-013-0588-8 24068389 PMC3966986

[5] Choko AT, Fielding K, Johnson CC, Kumwenda MK, Chilongosi R, Baggaley RC, et al. Partner-delivered HIV self-test kits with and without financial incentives in antenatal care and index patients with HIV in Malawi: a three-arm, cluster-randomised controlled trial. Lancet Glob Health. 2021;9(7):e977–88. doi: 10.1016/S2214-109X(21)00175-3 34143996 PMC8220130

[6] Kumar AKS, Chen LC, Choudhury M, Ganju S, Mahajan V, Sinha A, et al. Financing health care for all: challenges and opportunities. Lancet. 2011;377(9766):668–79. doi: 10.1016/S0140-6736(10)61884-3 21227490

[7] Branning G, Vater M. Healthcare Spending: Plenty of Blame to Go Around. Am Health Drug Benefits. 2016;9(8):445–7. 28465772 PMC5394555

[8] Chiang Y-S, Takahashi N. Network homophily and the evolution of the pay-it-forward reciprocity. PLoS One. 2011;6(12):e29188. doi: 10.1371/journal.pone.0029188 22195019 PMC3240652

[9] Hyde CR. Pay it forward: Simon and Schuster; 2014.

[10] Byrne M, Tan RKJ, Wu D, Marley G, Hlatshwako TG, Tao Y, et al. Prosocial Interventions and Health Outcomes: A Systematic Review and Meta-Analysis. JAMA Netw Open. 2023;6(12):e2346789. doi: 10.1001/jamanetworkopen.2023.46789 38064214 PMC10709779

[11] Li KT, Tang W, Wu D, Huang W, Wu F, Lee A, et al. Pay-it-forward strategy to enhance uptake of dual gonorrhea and chlamydia testing among men who have sex with men in China: a pragmatic, quasi-experimental study. Lancet Infect Dis. 2019;19(1):76–82. doi: 10.1016/S1473-3099(18)30556-5 30587296 PMC6347395

[12] Sung A, Zhang TP, Huang W, Tang W, Alexander M, Forastiere L, et al. Development of a Psychometric Tool to Measure Community Solidarity Among Sexual Minorities: Evidence From a Pay-it-Forward Randomized Controlled Trial. Sex Transm Dis. 2022;49(9):628–34. doi: 10.1097/OLQ.0000000000001659 35675708 PMC9378620

[13] Tang W, Xie Y, Xiong M, Wu D, Ong JJ, Wi TE, et al. A Pay-It-Forward Approach to Improve Chlamydia and Gonorrhea Testing Uptake Among Female Sex Workers in China: Venue-Based Superiority Cluster Randomized Controlled Trial. JMIR Public Health Surveill. 2023;9:e43772. doi: 10.2196/43772 36862485 PMC10020898

[14] Wu D, Jin C, Bessame K, Tang FF-Y, Ong JJ, Wang Z, et al. Effectiveness of a pay-it-forward intervention compared with user-paid vaccination to improve influenza vaccine uptake and community engagement among children and older adults in China: a quasi-experimental pragmatic trial. Lancet Infect Dis. 2022;22(10):1484–92. doi: 10.1016/S1473-3099(22)00346-2 35868342 PMC9492551

[15] Yang F, Zhang TP, Tang W, Ong JJ, Alexander M, Forastiere L, et al. Pay-it-forward gonorrhoea and chlamydia testing among men who have sex with men in China: a randomised controlled trial. Lancet Infect Dis. 2020;20(8):976–82. doi: 10.1016/S1473-3099(20)30172-9 32530426 PMC8957706

[16] Zhang Y, Li J, Xie Y, Wu D, Ong J, Marley G, et al. Pay-it-forward incentives for hepatitis virus testing in men who have sex with men: a cluster randomized trial. Nat Med. 2023;29(9):2241–7. doi: 10.1038/s41591-023-02519-w 37640859

[17] Marley G, Tan RKJ, Wu D, Wang T, Sun M, Sheng Q, et al. Pay-it-forward gonorrhea and chlamydia testing among men who have sex with men and male STD patients in China: the PIONEER pragmatic, cluster randomized controlled trial protocol. BMC Public Health. 2023;23(1):1182. doi: 10.1186/s12889-023-16095-8 37337181 PMC10280958

[18] Lu Y, Ni Y, Li X, He X, Huang S, Zhou Y, et al. Monetary incentives and peer referral in promoting digital network-based secondary distribution of HIV self-testing among men who have sex with men in China: study protocol for a three-arm randomized controlled trial. BMC Public Health. 2020;20(1):911. doi: 10.1186/s12889-020-09048-y 32532280 PMC7290069

[19] Braun V, Clarke V. Using thematic analysis in psychology. Qualitative Research in Psychology. 2006;3(2):77–101. doi: 10.1191/1478088706qp063oa

[20] Chapman CM, Louis WR, Masser BM, Thomas EF. Charitable Triad Theory: How donors, beneficiaries, and fundraisers influence charitable giving. Psychology and Marketing. 2022;39(9):1826–48. doi: 10.1002/mar.21701

[21] Choko AT, Fielding K, Johnson CC, Kumwenda MK, Chilongosi R, Baggaley RC, et al. Partner-delivered HIV self-test kits with and without financial incentives in antenatal care and index patients with HIV in Malawi: a three-arm, cluster-randomised controlled trial. Lancet Glob Health. 2021;9(7):e977–88. doi: 10.1016/S2214-109X(21)00175-3 34143996 PMC8220130

[22] Mizuno Y, Koenig LJ, Wilkes AL, Gelaude D, Carter J Jr, Scales White L, et al. Utilization of HIV Prevention, Care, and Treatment Services Among Young Men Who Have Sex With Men and Transgender Persons of Color in the U.S. South: A Qualitative Analysis. AIDS Educ Prev. 2022;34(6):512–27. doi: 10.1521/aeap.2022.34.6.512 36454137 PMC10986447

[23] Schmukle SC, Korndörfer M, Egloff B. No evidence that economic inequality moderates the effect of income on generosity. Proc Natl Acad Sci U S A. 2019;116(20):9790–5. doi: 10.1073/pnas.1807942116 31036660 PMC6525487

[24] Côté S, House J, Willer R. High economic inequality leads higher-income individuals to be less generous. Proc Natl Acad Sci U S A. 2015;112(52):15838–43. doi: 10.1073/pnas.1511536112 26598668 PMC4702979

[25] Macchia L, Whillans AV. The link between income, income inequality, and prosocial behavior around the world: A multiverse approach. Social Psychology. 2023;52(6):375–86.

[26] Hariwibowo IN, Wulandari CE, Setyohadi DB. Agency Relation in Online Charity Crowdfunding: The Role of Transparency to Attract Donation. IBIMABR. 2022:1–18. doi: 10.5171/2022.506046

[27] Andreoni JPIL-AG-V S-CKJMYE. Handbook of giving, reciprocity and altruism North-Holland: Elsevier. 2006:1201-69.

[28] Heiss SCaA. Dynamics of International Giving: How Heuristics Shape Individual Donor Preferences Nonprofit and Voluntary Sector Quarterly 50, no 3 (June 2021): 481–505.

