# Supplementary material for: Financing STI testing among men in China: A mixed-methods study of pay-it-forward monetary donations
Source: PLoS One. 2026 Feb 13;21(2):e0342595. doi: 10.1371/journal.pone.0342595 (PMC12904436; doi:10.1371/journal.pone.0342595)
Supplement: S1 File — (DOCX) [file pone.0342595.s001.docx]

***Appendix A. PIONEER Survey Instrument***

**PIONEER Survey Instrument**

**A. Sociodemographics**

*The next set of questions will ask you to provide some information about yourself.*

A1. Age: ____ years old

A2 What is your gender identity？

1. Male
2. Female
3. Non-binary
4. Gender neutral
5. Other________

A3. Current marital status:

1. Never married

2. Engaged or Married

3. Separated or divorced

4. Widowed

A4. Highest level of completed education:

1. Elementary

2. Middle school

3. High school or vocational school

4. Bachelor or associate degree

5. Above bachelor’s degree

A5. What is your total individual **monthly** income from all sources?

1. <1500 RMB/month

2. 1500-3000 RMB/month

3. 3001-5000 RMB/month

4. 5001-8000 RMB/month

5. >8000 RMB/month

A6. Who did you have sex within the past 12 months? Sex here refers to oral, anal, or vaginal intercourse.

1. Only women

2. Only men

3. Both men and women

**MSM Participants**

***B. Sexual behaviors***

*The next set of questions will ask you about your sexual behaviors with other men.*

B1. Your current male sexual partner(s) status is (select all that apply): ()

1. Stable (Long-term）
2. Casual
3. Both

B2. In the past 3 months, how many stable male sexual partners have you ever had? ______(numbers)

B3. In the past 3 months, how many casual male sexual partners have you ever had? ______(numbers)

B4. How do you typically find your male sexual partners? (Multiple)

1. Mutual friend

2. Brothel

3. Entertainment establishments (e.g. karaoke lounge, bar)

4. Social media (e.g. Blued)

5. Online forums or websites

6. Other, please specify _________

B5. Have you had sexual behaviors as follows:

|  | Yes | No |
| --- | --- | --- |
| Anal sex | ○ | ○ |
| Oral sex | ○ | ○ |
| Vaginal sex | ○ | ○ |

B6. What is your role during anal sex?

1. Mostly receptive (bottom)

2. Mostly insertive (top)

3. Half and half (versatile)

B7. In the past 3 months, when you had sex (oral, anal, or vaginal), how frequently did you use condoms?

1. Never

2. Sometimes

3. All the time

B8. In the past, have you told anyone about your sexuality or sexual history with men? (Select all that apply)

1. Yes, my long-term female partner/wife

2. Yes, my family members

3. Yes, my friends

1. Yes, my healthcare providers
2. Yes, others: _________

6. No one

**C. Clinical Information**

C1. The below asks about your history of STD testing according to disease::

|  | Never tested | Tested over a year ago | Tested in the past year |
| --- | --- | --- | --- |
| HIV* | ○ | ○ | ○ |
| Syphilis | ○ | ○ | ○ |
| Hepatitis C | ○ | ○ | ○ |
| Hepatitis B | ○ | ○ | ○ |
| Gonorrhea* | ○ | ○ | ○ |
| Chlamydia* | ○ | ○ | ○ |

**HIV: HIV (human immunodeficiency virus) is a virus that attacks the body's immune system. If HIV is not treated, it can lead to AIDS (acquired immunodeficiency syndrome).*

**Gonorrhea is a sexually transmitted disease (STD) caused by infection with the Neisseria gonorrhoeae bacterium.*

**Chlamydia is a common STD that can cause infection among both men and women.*

C2. Where did you get your last testing for STDs?

1. Self-testing
2. Hospital/local community health center
3. LGBTQ+organizations
4. Blood donation agencies
5. Centers for Disease Control and Prevention

C3. Where have you ever sought treatment for STDs? (Select all that apply)

1. No experience
2. Public hospitals
3. Private hospitals or private clinics
4. Chinese medicine hospitals or Chinese medicine clinics
5. Pharmacy
6. Other, please specify _________

C4. Have you ever diagnosed and treated yourself for an STD without consulting a physician?

1. Yes
2. No (skip to C6)

C5. Where did you get the medicine for the STD treatment? (Select all that apply)

1. No medicine treatment, symptoms vanished
2. Hospitals or pharmacies using previous prescriptions
3. Remaining medication from the latest treatment
4. Local pharmacies or online without prescriptions
5. Family, friends or others
6. Chinese medicine clinics
7. Other, please specify _________

C6. How much did you normally spend each time for sexually transmitted diseases testing?

1. free
2. less than 200 yuan
3. 200-500 yuan
4. 501-1000 yuan
5. more than 1000 yuan

***D. Community Engagement***

*The next set of questions asks about your experiences with LGBTQ+ community-related causes, events and organizations.*

D1. Have you ever participated in online forums or discussions on social media (ie. Weixin, Weibo, Twitter, or other online communities) about issues related to the LGBTQ+ community?

1. Yes
2. No

D2. Are you aware of any ongoing LGBTQ+ community related events？

1. Yes
2. No

D3. Have you ever encouraged others to use public health resource services, such as HIV/syphilis testing?

1. Yes
2. No

D4. Have you ever attended LGBTQ+ community related events?

1. Yes
2. No

D5. Have you ever donated to LGBTQ+ community related causes, events, or organizations? (other than today)

1. Yes
2. No

D6. Have you ever volunteered for LGBTQ+ community causes, events, or organizations?

1. Yes
2. No
3. Not sure, please specify ____________

1. **PIF Participation**

G1. Today, you get tested by:

1. Yourself (Skip To G3)
2. With a friend from the LGBTQ+ community
3. With a family member
4. Other___________

G2. How do you know this pay-it-forward?

1. At the hospital or clinic
2. Social media
3. LGBTQ+ community-based organizations
4. Friends from the LGBTQ+ community
5. Referral of medical staff
6. Other_______________

G3. What are your reasons for participating in the pay-it-forward project? (multiple choice)

1. To get a free test.
2. To receive incentive
3. To obtain an authoritative test result for health status
4. Received a testing invitation from a friend from the LGBTQ+ community
5. Trust in the community-based organization or clinics that introduced the project
6. Had an interest in this research project
7. The design of the pay-it-forward project is LGBTQ+ friendly
8. Other___________________

**Non-MSM participants**

**J. Sexual behaviors**

*The next set of questions will ask you about your sexual behaviors with women.*

J1. In the past 3 months, how many stable female sexual partners have you ever had? ______(numbers)

J2. In the past 3 months, how many casual female sexual partners have you ever had? ______(numbers)

J3. In the past 3 months, when you had sex, how frequently did you use condoms?

1. Never

2. Sometimes

3. All the time

J4. How do you typically find your sexual partners? (Multiple)

1. Everyday life (e.g. workplace, friends)

2. Brothel

2. Massage parlor

3. Entertainment establishments (e.g. karaoke lounge, bar, pub, nightclub)

4. Online dating applications (e.g. Tantan, Momo)

6. Social media

7. Online forums or websites

8. Other, please specify _________

**K. Clinical information**

K1. Before today, have you ever tested in the past (Select all that apply):

|  | Never tested | Tested over a year ago | Tested in the past year |
| --- | --- | --- | --- |
| HIV* | ○ | ○ | ○ |
| Syphilis | ○ | ○ | ○ |
| Hepatitis C | ○ | ○ | ○ |
| Hepatitis B | ○ | ○ | ○ |
| Gonorrhea* | ○ | ○ | ○ |
| Chlamydia* | ○ | ○ | ○ |

**HIV: HIV (human immunodeficiency virus) is a virus that attacks the body's immune system. If HIV is not treated, it can lead to AIDS (acquired immunodeficiency syndrome).*

**Gonorrhea is a sexually transmitted disease (STD) caused by infection with the Neisseria gonorrhoeae bacterium.*

**Chlamydia is a common STD that can cause infection among both men and women.*

K2. Where did you get your last testing for STDs?

1. Self-testing

2. Hospital/local community health center

3. Community organizations

4. Blood donation agencies

5. Centers for Disease Control and Prevention

K3. How much did you normally cost for sexually transmitted diseases testing per visit?

1. Free

2. less than 200 yuan

3. 200-500 yuan

4. 501-1000 yuan

5. more than 1000 yuan

K4. Which of the following factors do you think would influence you to get tested for STDs? (Select all that apply)

1. Time

2. Cost

3. Information about STDs and STDs testing

4. Fear of knowing having a severe disease after testing

5. Attitude and service of hospitals or medical institutions

6. Difficult to talk about

7. Other, please specify ________

**L. *Community Engagement***

*The next set of questions asks about your experiences with community-related causes, events and organizations.*

L1. Have you ever volunteered for any causes, events, or organizations? (other than today)

1. Yes

2. No

3. Not sure, please specify ____________

L2. Have you ever donated to LGBTQ+community related causes, events, or organizations? (other than today)

1. Yes

2. No

L3. Have you ever donated to other community related (non-LGBTQ+) causes, events, or organizations? (other than today)

1. Yes

2. No

L4. Have you ever participated in online forums or discussions on social media (ie. Weixin, Weibo, Twitter, or other online communities) about issues related to the LGBTQ+ community?

1. Yes

2. No

L5. Have you ever participated in online forums or discussions on social media (ie. Weixin, Weibo, Twitter, or other online communities) about issues related to the non-LGBTQ+ community?

1. Yes

2. No

**Experience and Feelings of Participation**

1. Your recruitment number ______________

1. The last four digits of your phone number ____________

1. What sample did you test today?
2. Self-service sampling
3. With the help of medical staff

1. Did you donate during participating PIF?(Only for PIF groups)
2. Yes (Skip to 4)
3. No (Skip to 5)

5. Your donation amount_______ (number)

6. Did you create a message, postcard, image, or other material to encourage others to receive testing?(only for PIF community)

a. Yes

b. No

7. Please fill in **THREE** words to describe how you felt immediately after you participate PIF

________ __________ _________

**New Gratitude Scale**

8. Do you agree or disagree with the following statement:

*Gratitude Scale: (Likert scale: 1= strongly disagree; 2= disagree; 3= slightly disagree; 4= neutral; 5=slightly agree; 6=agree; 7= strongly agree)*

1) I am thankful to the program for free testing.

2) I am thankful to the program for the opportunity to better understand my health situation.

3) I am thankful to the program for the opportunity to get connected with others.

4) My willingness to donate was because I wanted to express my kindness or goodwill.

5) After my participation in the program, I felt the care of others.

6) After my participation in the program, I felt a sense of warmth.

7) After my participation in the program, I am willing to introduce this program to others.

9. ***Donation related question***

1. Once I got to know that the donation came from others (knowing the local community cared about you and donated money for your test), it felt like an obligation to give back to others.
2. My reluctance to donate was because I hope to spend the money on myself, and not on others.
3. My reluctance to donate was because of my tight financial situation.

***Q. Project understanding***

Q1. According to your understanding, who funded the cost of your free CT/NG test?

1. Government and CDC
2. Research organization
3. Previous participants
4. Hospital or community organization
5. I don’t know

Q2. As far as you know, how will your donation be handled?

1. My donation will support a government or CDC initiative
2. My donation will only be used to support other participants’testing
3. My donation can be used by the organization for any purpose
4. I haven’t heard anything about donations
5. I don’t know where my donation will be used

Q3. What do you personally think are the benefits of the“Relay Inspection”project? (Multiple choice)

1. I can get free testing?
2. I can understand my STD infection status
3. If someone helps me, I can also help others.
4. We can help more community friends detect
5. Others: ____________________

Q4. What is your sexual orientation?(skip question)

1. Straight or heterosexual
2. Gay or homosexual
3. Bisexual
4. Pansexual
5. Asexual
6. Not sure / Undecided / Another identity not captured here
7. Other ____________

1. Community connectedness (Sexual minority only)

*(1=Strongly Disagree, 2=Disagree, 3=Agree, 4=Strongly Agree)*

S1. I feel that I am a part of the LGBTQ+ community.

S2. I feel a connection with other minority men in my community. *(New question)*

S3. I am willing to contribute to community activities for sexual minorities. *(New question)*

S4. Participating in the LGBTQ+ community is a positive thing for me.

S5. I am proud of the LGBTQ+ community.

S6. It is important to me to speak out for the rights related to the LGBTQ+ community.

S7. As long as I work with my peers in the LGBTQ+ community, the problems in the LGBTQ+ community we can be solved.

S8. I really feel that any problems faced by the LGBTQ+ community are also my own problems.

S9. If I need to discuss my personal issues with someone, I would be more inclined to talk to a friend from the LGBTQ+ community.

S10. If I needed a place to stay, I would be more inclined to seek out friends from the LGBTQ+ community to help.

S11. I can trust most of the LGBTQ+ people I know.

1. **Self-identification/Internalized Homophobia**

*(1=Strongly disagree, 2=Disagree, 3=Neither agree nor disagree, 4=Agree, 5=Strongly agree)*

T1. If possible, I would prefer to be heterosexual.

T2. If I were heterosexual, I would be happier.

T3. If there are some ways to change my sexual orientation, I would like to try.

T4. It is forbidden to reveal your sexual orientation in the civil service system.

T5. If you reveal your sexual orientation in the workplace, it will endanger your career.

T6. In most situations, I do care about whether other people know about my sexual orientation.

T7. I am worried that my sexual orientation will disgrace my family.

T8. Any mentions of the word “homosexuality” or “LGBTQ+” make me feel panic.

T9. I cannot fulfill traditional filial piety, which makes me feel impious.

1. **Community conectedness(straight-identified only)**

(*1=Strongly disagree, 2=Disagree, 3=Agree, 4=Strongly agree*)

U1. I feel a connection with other men in the community

U2. I am happy to contribute to community activities

U3. The problems faced by the community are also problems I need to face personally.

U4. As long as we work together, the problems facing the community can be solved.
