# Supplementary material for: Financing STI testing among men in China: A mixed-methods study of pay-it-forward monetary donations
Source: PLoS One. 2026 Feb 13;21(2):e0342595. doi: 10.1371/journal.pone.0342595 (PMC12904436; doi:10.1371/journal.pone.0342595)
Supplement: S2 File — (DOCX) [file pone.0342595.s002.docx]

**PIONEER Semi-Structured Interview Guide**

**Introduction:**

Thank you for participating in our interview. We hope to record this interview to avoid missing important information. If you do not want to be recorded, or if you want to suspend the recording during the process, please let us know at any time.

During the interview, we hope you can share your experience when participating in the "pay-it-forward" project, your thoughts before and after the test and participation in the study, and your specific feedback on all aspects of the project. You can choose to skip any questions in the interview, or you can suspend or terminate the interview at any time.

Do you have any questions now? If not, shall we start?

Do I have your permission to start recording?

**Basic information**

Let’s start with a few basic questions.

1. Socio-demographic information: First, we want to know some basic information about you.
   1. Which city do you live in?
   2. What is your current occupation?
2. Testing sites: We would also like to know about the testing site where you participated in the "pay-it-forward" project.
   1. Where did you participate in the "pay-it-forward" project? Are you familiar with this testing site? Are you familiar with the project implementer (doctor or nurse)?
   2. Have you tested for other STIs here before? If yes, where did you have the tests?

**"Pay-it-forward" concept understanding and project participation experience**

1. Introduction to inspection and "pay-it-forward" items
   1. Project impression: Can you tell us roughly your thoughts and impressions about the "pay-it-forward" project?
   2. What is your impression of the project implementer (doctor or nurse) who introduced the "pay-it-forward"?
2. Pay-it-forward: We would like your impression of "pay-it-forward" concept.
   1. What do you think about the "pay-it-forward" concept?
   2. Does pay-it-forward change how you think about testing?
   3. **(****OPTIONAL)** Did you visit the website and watch the video? Any other impressions?

[If no, please show them the website at the end of the interview and get their feedback for the sake of time]

1. Participation
   1. Which aspects of the project ultimately led to your decision to participate and have the tests?

[Try to ask: feeling the connection between yourself and the community? Recent risky behaviors? Discounted price?]

- 1. What do you think of your participation in the program? (Was it passing on your love? Obligation? Reciprocity? etc.)
  2. Is there any other way to promote your willingness to participate in the project? (e.g., mini program, notification about donation flow, and authorization from third-party institution)
  3. [Only for community-engaged PIF arm] Did you see the postcard written by the previous participants? Did you create a message, image, or text as part of the project?
  4. [Only for community-engaged PIF arm] Can these postcards help you better understand the "pay-it-forward" project?
  5. [Only for community-engaged PIF arm] If you did, what did you write/share and what was your inspiration for this message?
  6. [Only for community-engaged PIF arm] Any other activities that you can suggest to promote participation and increase interaction?

1. **(OPTIONAL)** Trust and hesitation
   1. Do you feel that you were able to confide in this service provider and trust that he/she would not discuss your health concerns with others?
   2. What aspects make you trust the "pay-it-forward" project?
   3. What aspects make you worried or hesitant about participating in the “pay-it-forward” project?

[Try to ask: the gender of the project executor, the sample collection process, direct payment or donation by scanning the QR code, the time to obtain the test results, such as the results found to be positive, follow-up services, etc.]

**Sexual behavior, follow-up, and partner notification**

To help us design better services in the future, we would like to ask you a bit about your sex partners and your experiences of follow-up. Some of these questions may be sensitive, and you can decline to answer at any time.

Status of sexual partners:

1. Can you tell us about your sex partners from the time of the test?
   1. [Follow-up] Do you have a stable partner when you received the test?
   2. Is your partner female or male?
   3. Have you ever purchased sex before?
   4. If yes, how did you find female/male sex workers? Did you wear protective measures such as condoms during sex?
2. Did you test regularly for STIs? How often?
3. Did your partner come with you for testing? Would you prefer to test yourself or testing with a partner? Why?
4. How does having a partner affect your decision on whether to get tested?
   1. [Follow-up] Does having a partner make you more likely or less likely to get tested?
   2. [Follow-up] What scenarios or characteristics of you and your partner might make you more or less likely to get tested?
   3. Prior to getting tested, did you think at all about what you would do if you tested positive? Did this influence your decision on whether to test?

Sexual health services:

1. How have you been referred to/accessed sexual health services?
2. What are the barriers and facilitators for you to access sexual health services?
   1. What make you want to visit sexual health clinic?

[Try to probe on: Fear towards STI, high-risk sexual behavior, self-consciousness in genital examination, trust in health professionals, etc]

- 1. Any other aspects that prevent you from accessing services or seeing a doctor?

[Try to probe on: Cost, physician attitude, physician gender, perceiving STIs as not serious, stigma, etc]

1. What do you think are the best and worst aspects of attending STI testing services in the public clinics?

[Try to ask about these aspects: Cost, access/appointments, waiting time, privacy/confidentiality, location and clinic environment, support, etc]

1. What problems/difficulties have you faced in using services/getting results/treatments?
   1. Did this happen each time you accessed services?
   2. Do you think this is a particular issue for this group? (i.e. men, gay people, straight people, younger/older people)
2. How do you think STI testing services could be offered differently or improved?

[Positive testers only] Treatment and PN:

1. Did you test positive for either CT or NG? [Proceed only if yes]
2. Did you get treated?
   1. If yes, where did you get treated? Can you tell us about your experience?
   2. If no, why not? What were the main barriers to getting treated?
3. Did you have to follow up with another appt to get retested?
   1. If yes, can you tell us about this experience?
4. How do you think treatment process could be improved? (Some examples might include: Having an appointment time without having to wait in line; Getting medications from a pharmacy without having to see a doctor)
5. Did you tell any of your sex partners about your positive test? Why or why not?
   1. How did you decide whether to tell them, or which ones to tell?
   2. What are the main barriers to telling your partners about your positive test?
   3. Does your decision to tell your partner change based on your relationship with this partner? Please explain.
6. Did any of your sex partners get tested or treated? If yes, how was their experience?
7. What do you think could be done to facilitate telling different types of partners, either stable partners or casual partners, if you get a positive test? (Some examples might include, Notification card or WeChat notification post to share, a testing kit that your partners could take and then mail back, or directly delivering medications to your partners)

**Donation**

**[FOR PEOPLE WHO DID NOT DONATE]**

- - - 1. How did you feel when you were asked you to donate?
      2. Why did you choose to not donate at that time? What are the potential factors that influence your decision? (Possible reasons include: the failure to understand PIF concept, the implementer didn’t present the donation QR code)

**[FOR PEOPLE WHO DONATED]**

Think back to the amount of money you donated to the “pay-it-forward” project:

- - - 1. How much did you donate? (Check the survey record before the interview)
      2. Why did you choose to donate this amount? What factors might make you donate more/less?
      3. Compared with your peers, how do you feel about the amount of your donation?
      4. What do you think is the average donation amount for the "pay-it-forward" project?
      5. How did you feel when you were asked you to donate?
      6. Mechanism for why people donate. We observe that there is a mix of emotional and practical reasons to donate. What do you think about the reason to receive the test?

[Feasible follow-up questions for donation: Dig at least two or three points based on the participants' answers.]

- For instance, some friends who may benefit from testing, feel that helping others to test can protect themselves from STDs and feel that they can contribute to the gay community, want to accumulate merit, or have to pay because of peer pressure, etc.
- What do you think of this idea if there are some people who have higher incomes and let them pay for the testing of people with lower incomes?
- If you could donate to an individual person or to a group, would that change how much you would be willing to donate?
- If you could donate to CDC, government, or public clinic, would that change your willing to donate and how much you would be willing to donate?

**"Pay-it-forward" sustainability**

1. Do you think whether the "pay-it-forward" project can be sustained after its completion? What aspects make it sustainable or not?
2. What are the problems that you can think about in sustaining "pay-it-forward"? How should we address these problems? [Try to ask about legal or financial aspects]
3. Do you believe that public clinics and hospitals can carry on with the "pay-it-forward" project? In what other contexts do you see the "pay-it-forward" project sustaining?

[Feasible follow-up questions for sustainment: Dig at least one or two points based on the participants' answers.]

- In your opinion, what is the impression of the male STD patient community in Guangdong Province about the pay-it-forward project?
- What other ideas do you have to improve the sustainment of "pay-it-forward"?

**[MSM only]**

**Skip the remaining questions if the participant is not identified as MSM or may feel uncomfortable.**

**MSM community and social context**

This PIF project was created for the health benefits of local MSM community in Guangdong Province, so we would like to know more about the gay community and hear your opinions.

- - - 1. MSM community:
         1. How is your connection with the gay community?
         2. How would you call your gay community in the language you prefer? [Maybe Nantong 男同, *Quanzi* 圈子, *Tongzhi* 同志，*Tongxunlu* 通讯录，partner*伴侣*, etc] Does the word “sexual minority” (性少数) or “LGBTQ” resonate with you?

[Follow up: Did you post on social media issues related to homosexuality or sexual health? Have you received other gay-oriented services before the test? Do your friends obtain relevant knowledge through you? Have you brought a friend for testing before? Have you ever been a volunteer in a gay-oriented organization?]

- - - - 1. What do you think of the gay community in your city? Is it active? How does it compare to the gay communities in other cities in Guangdong?
        2. Do you feel there are members in the gay community that you would be able to confide in that would want to listen?
        3. What are you most interested in the broad gay and LGBTQ topics?
      1. PIF and community engagement:
         1. Have you volunteered for other MSM or gay groups in your community?
         2. Do you want to help other local MSM? Is pay-it-forward a way to help other MSM? (Try to mention “互帮互助”mutual aid and mutual support)
         3. Did you participate in other MSM testing services and MSM research studies before? If yes, how is this project similar or different from them?
         4. Do you want to help other MSM while not being outed as gay? Or do you want to help other MSM while opening your gay identity?

**Other health services for MSM:** We are also interested in expanding health services for MSM.

- 1. Do you think that we did enough to improve MSM health? Are you concerned about the health of members of the LGBTQ community? Do you think there is any urgent issue that need to be fixed/addressed? What are some other important health services for you personally that you would like to see offered?
  2. What are the main challenges to accessing these services? [Eg cost, availability of PrEP, not wanting to see a doctor – probe further for any of these]
  3. We are interested in your thoughts about some different models for designing other health services for MSM. What do you think about the following: (for both, suggest at-cost as well as low-cost)
     1. Incorporating a doctor at CBOs once a week who can prescribe appropriate testing, medications including PrEP, and vaccines.
     2. Online access to a physician who can do telemedicine visits for testing, medications such as PrEP, with CBOs managing the testing and PrEP delivery.
     3. Being referred by CBOs to a local, MSM-friendly clinic for STI care, PrEP, and vaccines
  4. What other ideas or suggestions do you have for expanding health services for MSM?

​**Conclusion: Do you have any other questions or related suggestions?**
